# *Macaca fascicularis* and *Macaca nemestrina* infected with zoonotic malaria parasites are widely distributed in Sarawak, Malaysian Borneo

**DOI:** 10.1038/s41598-022-14560-9

**Published:** 2022-06-21

**Authors:** Thamayanthi Nada-Raja, Khamisah A. Kadir, Paul C. S. Divis, Dayang S. A. Mohamad, Asmad Matusop, Balbir Singh

**Affiliations:** 1 Malaria Research Centre, Faculty of Medicine and Health Sciences, Universiti Malaysia Sarawak, 94300 Kota Samarahan, Sarawak, Malaysia; 2 Sarawak State Health Department, 93050 Kuching, Sarawak, Malaysia

**Keywords:** Malaria, Epidemiology

## Abstract

Human infections with *Plasmodium knowlesi*, a malaria parasite of *Macaca fascicularis* and *Macaca nemestrina* (long-tailed and pig-tailed macaques respectively), occur throughout Southeast Asia, especially Malaysian Borneo. Other naturally-acquired human infections with malaria parasites from macaques in Southeast Asia are *P. cynomolgi, P. inui*-like, *P. coatneyi* and *P. simiovale*. In Sarawak, Malaysian Borneo, *M. fascicularis* and *M. nemestrina* from only the Kapit Division have been examined previously for malaria parasites. In order to determine the distribution of *P. knowlesi* and other zoonotic malaria parasites, 73 macaque blood samples derived from 7 other administrative divisions in Sarawak were studied. Of 45 blood samples from *M. fascicularis* and 28 from *M. nemestrina* tested by nested PCR assays, 23 (51.1%) *M. fascicularis* and 15 (53.6%) *M. nemestrina* samples were positive for *Plasmodium* DNA. Thirty-two of these macaques from 7 divisions sampled, harboured either single (*n* = 12), double (*n* = 9), triple (*n* = 7) or quadruple (*n* = 4) infections of *P. knowlesi*, *P. inui*, *P. cynomolgi* and *P. coatneyi*, while the infecting species of *Plasmodium* could not be identified for 6 samples. *P. knowlesi* was detected in 15.5% (7/45) *M. fascicularis* and in 7.1% (2/28) *M. nemestrina* sampled*.* Despite the small number of samples analysed from each administrative division, the current study indicates that macaques infected with the zoonotic malaria parasites *P. knowlesi, P. cynomolgi*, *P. inui* and *P. coatneyi* are widely distributed throughout Sarawak, Malaysian Borneo. Travelers to forested areas in Sarawak should be made aware of the potential risk of acquiring zoonotic malaria.

## Introduction

Non-human primates are reservoir hosts for malaria parasites and many other infective agents including Hepatitis B, Simian retroviruses, Macacine herpesvirus 1 and *Brugia malayi*^1, 2^. There are more than 30 species of *Plasmodium* which are capable of infecting primates out of over 200 species of *Plasmodium* identified to date^3, 4^. Human infections with simian malaria parasites were thought to be extremely rare until a large focus of human infections with *P. knowlesi* was reported in 2004 in the Kapit division of Sarawak, Malaysian Borneo^5^. Since then, human *P. knowlesi* infections have been reported throughout Malaysia and in all countries in Southeast Asia except Timor Leste^6–14^. Human knowlesi malaria cases are of public health concern in Malaysia, where from 2018 to 2020 they constituted all the 8,500 indigenous cases of malaria, mainly in the states of Sabah and Sarawak, Malaysian Borneo (Ministry of Health Malaysia, unpublished data)^15, 16^. In Sarawak, knowlesi malaria cases have continued to increase from the 120 cases first described in the Kapit Division in 2004 to between 759 and 1,247 annual cases from 2016 to 2020, with 14 deaths (Ministry of Health Malaysia, unpublished data). There have also been case reports of tourists acquiring knowlesi malaria following visits to forested areas in Sarawak^17–19^ and Southeast Asia^20^.

The natural hosts for *P. knowlesi* are primarily *Macaca fascicularis* (long-tailed macaque) and *M. nemestrina* (pig-tailed macaque)^4^. *P. knowlesi* infections in these species, the most common non-human primates in Southeast Asia, have been reported from Thailand, Malaysia, Philippines and Laos^7, 21–26^. There have also been reports of natural infections of *P. knowlesi* in a *Presbytis melalophos* (banded leaf monkey) in Peninsular Malaysia^27^, and in *Trachypithecus obscurus* (dusky leaf monkey) and *Macaca arctoides* (stump-tailed macaque) in Thailand^26, 28^. Apart from *P. knowlesi*, there are other malaria parasites of *M. fascicularis* and *M. nemestrina* which can infect humans. A case of naturally-acquired *P. cynomolgi* was reported in Peninsular Malaysia in 2014^29^. This was followed by descriptions of other human *P. cynomolgi* infections in Cambodia, Malaysia and Thailand^30–33^. More recently, human infections with *P. inui, P. coatneyi, P. simiovale* and *P. inui-*like parasites have been described in Malaysia^34, 35^. In South America, zoonotic malaria cases originating from other non-human primates have been reported; *P. brasilianum* acquired from *Alouatta seniculus* (red howler monkey)^36^ and *P. simium* from *Alouatta guariba* (brown howler monkey)^37, 38^.

Previous studies on malaria parasites of *M. fascicularis* and *M. nemestrina* in Sarawak State were confined to the Kapit Division^21, 39^. In order to determine the distribution of *P. knowlesi* and other zoonotic malaria parasites, macaque blood samples collected from 7 other administrative divisions in Sarawak were examined with molecular detection assays.

## Methods

### Ethics statement and collection of blood samples

In Sarawak, permits are required from the Sarawak Forestry Department and the Sarawak Biodiversity Centre for the collection of samples from animals and for the use of these samples for research. The protocols for capture, collection of blood samples and release of wild macaques, together with collection of blood samples from captive macaques were approved by the Sarawak Forestry Department (Permits Numbers: NPW.907.4.2-32, NPW.907.4.2-97, NPW.907.4.2-98, 57/2006 and 70/2007). In addition, a permit to access and collect animal blood samples was obtained from the Sarawak Biodiversity Centre (Permit Number: SBC-RP-0081-BS). All methods were performed in accordance with relevant guidelines and regulations.

A veterinarian collected blood samples from the femoral vein of each wild macaque following anaesthesia by intramuscular injection of tiletamine and zolazepam (Zoletil® 100, Virbac, France) at a dose of 5 mg/kg. The blood collection was performed using 21G × 1” eedle attached to a 5 ml syringe (Terumo, Japan), where not more than 5 ml blood was drawn from each macaque for the study purposes. Blood samples were collected at the trap sites and the animals were released after they had recovered from anaesthesia. Similar procedures were employed when venous blood samples were obtained from captive macaques at Matang Wildlife Centre and Semenggoh Wildlife Rehabilitation Centre in the Kuching division. For macaques kept as pets, blood spots on filter paper were collected by pricking the end of the tails of the animals by staff of the Sarawak Health Department during their visits to malaria affected communities. This study was approved by the Medical Ethics Committee of Universiti Malaysia Sarawak and was performed in concordance with the Animal Research: Reporting of In Vivo Experiments (ARRIVE) guidelines.

**Figure 1:**
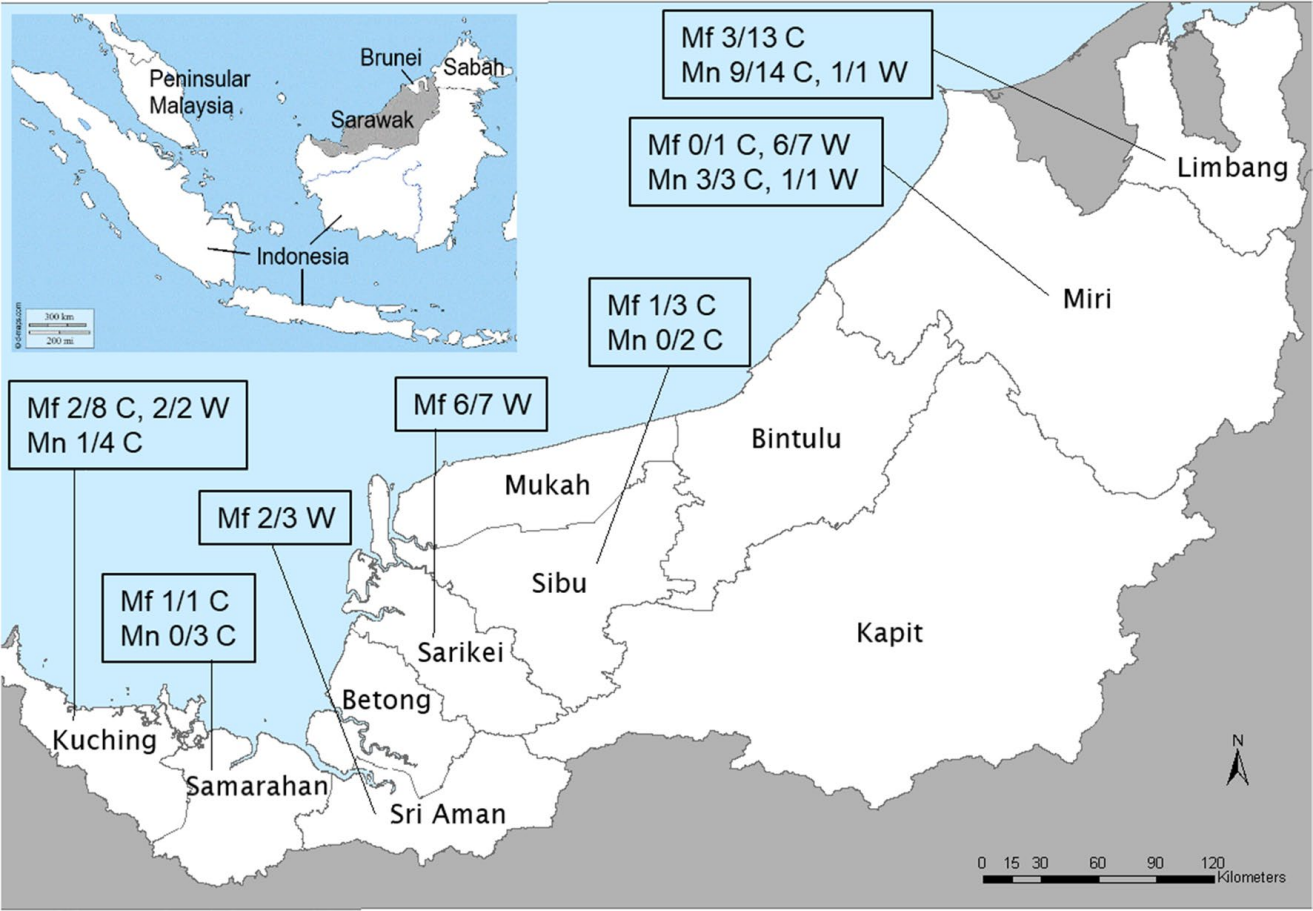
Prevalence of *Plasmodium* among macaques in the different administrative divisions in Sarawak, Malaysian Borneo. The map of Sarawak was adapted from Lee et al.^40^ and modified using Paint 3D app. The inset map showing the position of Sarawak (shaded) on the island of Borneo was obtained from https://d-maps.com/carte.php?num_car=28850&lang=en and modified using Paint 3D app. Mf = *M. fascicularis*, M*n* = *M. nemestrina*, Number *Plasmodium*-positive / Number tested, C = captive, W = wild. All the captive macaques, except those at the wildlife centres in the Kuching division, were kept as pets.

Blood samples were obtained from 45 M*. fascicularis* and 28 M*. nemestrina* that were either wild (*n* = 21) or captive (*n* = 52). The wild macaques were sampled at the following administrative divisions in Sarawak, with the number collected and dates of sampling in brackets: Sarikei (*n* = 7, January 2007 to February 2008), Kuching (*n* = 2, June 2008), Limbang (*n* = 1, June 2008), Miri (*n* = 8, June 2007 to June 2008) and Sri Aman (*n* = 3, September 2012 to October 2012) (Fig. 1). Samples from 12 captive macaques were obtained from the Kuching Division; 10 at the Matang Wildlife Centre from October 2003 to August 2012 and 2 from Semenggoh Wildlife Rehabilitation Centre in October 2004. The remaining 40 blood samples were from captive macaques that had been kept as pets by residents of rural communities in the following divisions, with the number collected and dates of sampling in brackets: Sibu (*n* = 5, June 2007 to July 2007), Samarahan (*n* = 4, June 2007 to July 2007), Limbang (*n* = 27, October 2003 to July 2008), Miri (*n* = 4, May 2007 to June 2007). The macaques at the wildlife centres were not born in captivity and were kept in enclosures that were not netted. No information was collected regarding the period of captivity of the macaques at the wildlife centres or at the rural communities. Each of the wild and captive macaques was only sampled once. The macaques were identified by the veterinarians and by the staff of the Sarawak Health Department based on the length of the tails of the macaques; *M. fascicularis* have long tails (32–75 cm) and *M. nemestrina* have very short tails (< 25 cm). The veterinarians at the wildlife centres were informed which macaques were malaria-positive and were advised to treat them with antimalarial drugs. None of the wild macaques that were trapped nor those kept as pets were treated with antimalarial drugs.

### DNA extraction

DNA was extracted from dried blood spots on filter paper using InstaGene Matrix (BioRad Laboratories) and from frozen blood samples using the QIAamp DNA Micro Kit (QIAGEN) as described previously^41^.

### Nested PCR assays

DNA samples were initially analysed using nested-PCR assays for detecting *Plasmodium* DNA^42^. All *Plasmodium*-positive samples were then analysed by nested PCR assays with PCR primers specific for *P. knowlesi*, *P. cynomolgi*, *P. inui, P. fieldi* and *P. coatneyi* as described previously^21^.

## Results

Of the 73 macaque blood samples examined with nested PCR assays, 51.1% (23/45) *M. fascicularis* and 53.6% (15/28) *M. nemestrina*) were positive for *Plasmodium* DNA (Fig. 1). The prevalence of malaria parasites was higher in wild than in captive *M. fascicularis*; 84.2% (16/19) of wild *M. fascicularis* and 26.9% (7/26) of captive ones were *Plasmodium-*positive. For *M. nemestrina*, both samples from the wild and 50% (13/26) of those from captive animals were *Plasmodium*-positive.

The 23 *Plasmodium*-positive *M. fascicularis* samples were collected from all the 7 administrative divisions sampled (Fig. 1). For *M. nemestrina*, 20 samples were collected for analysis from 5 administrative divisions and 15 macaques from Limbang, Kuching, and Miri divisions harboured malaria parasites while 5 from Sibu and Samarahan were found to be *Plasmodium*-negative.

In 84% (32/38) *Plasmodium*-positive samples from macaques, *P. knowlesi*, *P. inui*, *P. cynomolgi*, and *P. coatneyi* were detected either as single (*n* = 12), double (*n* = 9), triple (*n* = 7) or quadruple (*n* = 4) infections (Table 1). *P. knowlesi* was detected in 15.5% (7/45) *M. fascicularis* and in 7.1% (2/28) *M. nemestrina* sampled*.* For 6 of these samples (3 each from *M. fascicularis* and *M. nemestrina),* the species of *Plasmodium* could not be identified with PCR primers specific for *P. knowlesi*, *P. cynomolgi*, *P. inui, P. fieldi* and *P. coatneyi*.

**Table 1:** Results of species-specific PCR assays for *Plasmodium-*positive macaques.

NHP	Captive/wild	Number positive													
		Pk	Pct	Pcy	Pin	Pk + Pin	Pin + Pct	Pin + Pcy	Pcy + Pct	Pk + Pcy + Pct	Pk + Pin + Pct	Pcy + Pin + Pct	Pk + Pcy + Pin + Pct	*Could not be identified	Total
Mf	Captive	0	1	0	0	1	1	0	0	0	1	0	1	2	7
	Wild	1	0	0	1	0	3	1	1	1	1	3	3	1	16
Mn	Captive	1	0	2	6	1	0	1	0	0	0	0	0	2	13
	Wild	0	0	0	0	0	0	0	0	0	0	1	0	1	2
Total		2	1	2	7	2	4	2	1	1	2	4	4	6	38

*NHP*: Non-human primates, *Mf*: *M. fascicularis*, *Mn*: *M. nemestrina*, *Pk*: *P. knowlesi*, *Pct*: *P. coatneyi*, *Pcy*: *P. cynomolgi*, *Pin*: *P. inui.*

*Samples were *Plasmodium*-positive but species of *Plasmodium* could not be identified with primers specific for *P. knowlesi*, *P. cynomolgi*, *P. inui, P. fieldi* and *P. coatneyi.*

## Discussion

Molecular detection methods such as nested PCR assays have proved to be more sensitive and specific than microscopy for the detection of malaria parasites in humans, non-human primates and mosquitoes^5, 7, 23, 39, 43–46^. In the current study, for 6 macaque samples that were *Plasmodium*-positive in nested PCR assays, the species of *Plasmodium* could not be identified with nested PCR assays using species-specific primers for *P. knowlesi*, *P. cynomolgi*, *P. inui, P. fieldi* and *P. coatneyi.* Similar observations have been reported in studies of blood samples from macaques^28, 47^ and humans^34^, and during entomological studies to incriminate vectors of malaria^45–47^. For these 6 samples the approximately 240 bp fragments that were amplified by PCR with the *Plasmodium*-specific primers, rPLU3 and rPLU4, were not sequenced. Previously, we sequenced these fragments from a number of samples that were *Plasmodium*-positive but negative with the species-specific PCR primers and found through the Basic Local Alignment Search Tool (BLAST) searches that they had high similarity to species of *Plasmodium*, including *P. inui* and *P. knowlesi*. However, it was not possible to confirm the identity of the infecting species by phylogenetic analyses of these and referral sequences since the bootstrap values were low and it was not possible to differentiate between the various species of *Plasmodium*. It is possible that the parasite counts in these 6 macaques were low but were sufficiently amplified by the genus-specific primers which target all the copies of both the A- and S-forms of the small subunit (SSU) rRNA genes^41, 42^. However, with the species-specific primers that target either the S- or the A-type SSU rRNA genes, sufficient PCR amplification was probably not attained and no amplicons were detected by gel electrophoresis. An alternative explanation is that these macaques may have been infected by *P. simiovale*, which has been demonstrated in *M. fascicularis* in Sarawak^39^, and/or by one or more species of *Plasmodium* for which PCR primers are currently unavailable.

The complex nature of infections with *Plasmodium* was noted in the current study where 84.2% (32/38) of the *Plasmodium*-positive macaques had either double, triple or quadruple infections with *P. knowlesi, P. inui, P. cynomolgi* and *P. coatneyi*. Similar observations were made in our previous study in the Kapit Division of Sarawak where 101 of 108 wild macaques were *Plasmodium*-positive and 91% (91/101) had mixed species infections^21^. Multiple species of *Plasmodium* in each macaque host, as reported in that^21^ and other studies^28, 39^, may not accurately reflect the actual number of species of *Plasmodium* harboured by each macaque, since blood samples at only a single time point were obtained and analysed. It has been noted that parasite counts fluctuate and can even change daily in non-human primates that have been kept in captivity^4^. In addition, erythrocytes containing late asexual stages of certain parasites like *P. coatneyi* sequester^4^, so the infection would not be detected by PCR analysis of blood samples if the sample was collected when the majority of the parasites were late stages. Sequential samples would provide more accurate information on the prevalence of each species of *Plasmodium* in a host in nature but it would not be possible to undertake these investigations on wild macaques due to ethical and logistic reasons.

It is important to determine the geographical range of *M. fascicularis* and *M. nemestrina* infected with zoonotic malaria parasites to inform the public of the risks of acquiring zoonotic malaria. Previous studies in Sarawak were confined to the Kapit Division and in the current study macaques from another 7 divisions were examined^21, 39^. A total of 32 macaques infected with either single or multiple species of the zoonotic malaria parasites *P. knowlesi, P. cynomolgi, P. inui* and *P. coatneyi* were found in all the 7 administrative divisions of Sarawak studied, from the southernmost division of Kuching to the northernmost division of Limbang. A limitation of the current study is the relatively small number of samples examined, involving between one to 14 macaques from each administrative division. There is a need for more extensive studies, involving a larger number of samples from varied locations at each administrative division to determine accurately the prevalence of malaria infection among macaques at these locations. Furthermore, these studies should focus on sampling wild macaques rather than captive ones. In the current study, the prevalence of malaria was much higher in wild than in captive macaques. This difference is primarily due to the mobility of the macaques, greater abundance of the mosquito vectors of *P. knowlesi* and other simian malaria parasites in the forests rather than close to human habitats, and to the feeding behaviour of the vectors. The vectors of *P. knowlesi* in Sarawak, Sabah, Peninsular Malaysia and Vietnam are forest-dwelling mosquitoes and a study in Sarawak, found that the vector, *Anopheles latens,* feeds predominantly in the forest and the forest fringe, rather than in longhouse communities^48, 49^. Due to the forest habitats and feeding behaviour of the vectors, wild macaques would be at risk of acquiring malaria while foraging for food or while resting at night on trees in the forest. In contrast, some of the captive macaques in the current study were housed in wildlife centres situated away from malaria transmission areas, and those kept as pets by residents of rural communities would have limited exposure to mosquito vectors as they were tethered. Therefore, examination of blood samples from wild as opposed to captive macaques would provide more accurate information on zoonotic malaria parasites that are present at areas surrounding each sampling site. Such information would be essential for informing the local population and visitors to rural areas of Sarawak of the risks of acquiring zoonotic malaria. Combined with data from concurrent entomological and community-based studies, they would provide valuable information on malaria transmission and assist in the implementation of appropriate prevention and control measures for zoonotic malaria.

In conclusion, despite the small number of macaques sampled at each administrative division, the current study indicates that that the zoonotic malaria parasites *P. knowlesi, P. cynomolgi, P. inui* and *P. coatneyi*, are found in *M. fascicularis* and *M. nemestrina* that are widely distributed throughout Sarawak, Malaysian Borneo. *P. knowlesi* is the main cause of malaria in Sarawak and has the potential to cause fatalities^5, 15, 44, 50, 51^ so the local rural population and travelers to habitats of these macaques in Sarawak should be made aware of the potential risk of acquiring *P. knowlesi* and other zoonotic malaria parasites.

## Data Availability

The datasets generated during and/or analysed during the current study are available from the corresponding author on reasonable request.
